# Assessment of the Sensitivity of Some Plant Pathogenic Fungi to 6-Demethylmevinolin, a Putative Natural Sensitizer Able to Help Overcoming the Fungicide Resistance of Plant Pathogens

**DOI:** 10.3390/antibiotics9120842

**Published:** 2020-11-25

**Authors:** Larisa Shcherbakova, Maksim Kartashov, Natalia Statsyuk, Tatyana Pasechnik, Vitaly Dzhavakhiya

**Affiliations:** All-Russian Research Institute of Phytopathology, Institute str., vl. 5, Bolshie Vyazemy, Moscow 143050, Russia; maki505@mail.ru (M.K.); beefarmer@yandex.ru (T.P.); vitaly@vniif.ru (V.D.)

**Keywords:** chemosensitization, antifungal compounds, plant pathogenic fungi, fungicide resistance, 6-demethylmevinolin, environmental pollution

## Abstract

Agricultural fungicides contaminate the environment and promote the spread of fungicide-resistant strains of pathogenic fungi. The enhancement of pathogen sensitivity to these pesticides using chemosensitizers allows the reducing of fungicide dosages without a decrease in their efficiency. Using Petri plate and microplate bioassays, 6-demethylmevinolin (6-DMM), a putative sensitizer of a microbial origin, was shown to affect both colony growth and conidial germination of *Alternaria solani*, *A. alternata*, *Parastagonospora nodorum*, *Rhizoctonia solani*, and four *Fusarium* species (*F. avenaceum, F. culmorum*, *F. oxysporum*, *F. graminearum*) forming a wheat root rot complex together with *B. sorokiniana*. Non- or marginally toxic 6-DMM concentrations suitable for sensitizing effect were determined by the probit analysis. The range of determined concentrations confirmed a possibility of using 6-DMM as a putative sensitizer for the whole complex of root rot agents, other cereal pathogens (*A. alternata*, *P.* *nodorum*), and some potato *(R. solani*, *A. solani)* and tomato (*A. solani*) pathogens. Despite the different sensitivities of the eight tested pathogens, 6-DMM lacked specificity to fungi and possessed a mild antimycotic activity that is typical of other known pathogen-sensitizing agents. The pilot evaluation of the 6-DMM sensitizing first confirmed a principal possibility of using it for the sensitization of *B. sorokiniana* and *R. solani* to triazole- and strobilurin-based fungicides, respectively.

## 1. Introduction

To meet an increasing demand for crop products, high-yielding cultivars of agricultural plants are now grown all over the world. One of the main conditions for fully realizing a potential productivity of such cultivars is a successful control of crop pathogens, primarily fungi, which may cause diseases resulting in up to 70–80% of yield losses [1]. In many countries, including Russia, causative agents of foot/root rots (*Fusarium* spp., *Bipolaris* spp., *Rhizoctonia* spp.), leaf and/or glume blotches (*Bipolaris* spp., *Alternaria* spp., *Parastagonospora* spp.) head blight, kernel smudge (*Fusarium* spp., *Alternaria* spp.), and wilt *(F. oxysporum*) belong to the most widespread and detrimental pathogens of such economically important crops as cereals, potato and tomato [2,3,4]. As in the case of other plant pathogenic fungi, a common practice to control these agents, and thus efficiently prevent significant yield losses caused by these pathogens, is the use of chemical fungicides. However, like medical antibiotics and antimycotics, the effectiveness of agricultural fungicides is under threat because of the evolution of fungicide resistance, which is often developed soon after a new fungicide is introduced due to improper and/or extensive fungicide application practices [5,6]. A wide use of modern fungicides resulted in a significant increase in the frequency of high fungicide resistance and multiple or cross-resistance of various plant pathogenic fungi. Fungicides with a single-site mode of action, such as Quinone outside Inhibitors or DeMethylation Inhibitors (QoI- and DMI-fungicides, respectively) commonly used against above-mentioned pathogens can reduce or lose their protective efficacy relatively quickly [7,8,9,10,11,12]. Moreover, in some cases, the time required for the emergence of resistant strains since the start of a commercial application of such fungicides do not exceed several years [13]. The attempts to control resistant forms by increasing the dosages or frequency of fungicide applications increase the total costs for plant protection and only complicate the problem resulting in an enhanced accumulation of such forms and stimulating their spreading across populations [14]. Apart from this problem, the excess of fungicides spread into the environment causing significant contamination of terrestrial and water ecosystems and multiple negative effects on soil microbiota, insects, vertebrates, as well as in poisoning of food and feed [15,16,17]. Thus, the solving of a fungicide resistance problem and associated problems of the excess fungicide applications and environment pollution become one of the dominant trends in the current plant protection science. 

Different strategies are proposed for agricultural practices to prevent or minimize negative side effects of extensive fungicidal treatments accompanied with increasing resistance of fungi and environmental risks [18,19]. A promising new approach intended to reduce fungicidal dosages without any mitigation of the antifungal effect is the enhancement of a pathogen sensitivity to fungicides (chemosensitization). This can be accomplished by the co-application of a fungicide with certain natural or chemical eco-friendly compounds (sensitizers) at concentrations, which should meet the following requirements. First, both fungicide and sensitizer, applied alone, should provide no or insignificant fungitoxic effect. Second, both compounds applied together should effectively suppress a target pathogen, preferably in a synergistic or in some cases in an additive manner [20,21]. The synergy between ineffective doses of the components occurs since a sensitizer and a fungicide attack different pathways of fungal metabolism or distant stages of the same metabolic pathway.

The chemosensitization approach was initially developed in medicine to overcome the resistance of pathogenic fungal hospital strains to antimycotic drugs. To date, a number of secondary plant metabolites, as well as their synthetic analogues, which can be used as chemosensitizing agents against human-infecting fungi, have been revealed. Some of them possess an antifungal activity, but significantly less than that of commercial antimycotics [20]. In contrast, only a few chemosensitization studies were recently performed for agricultural purposes [21,22,23], with a confirmed effect on plants in some cases [22,23]. The cited researchers demonstrated that not only plants but also bacteria [22] or filamentous fungi [23,24,25] may serve as the sources of metabolites significantly enhancing the sensitivity of plant pathogens to agricultural fungicides including such widely-used and rather persistent ones as triazoles [26]. 

The first research step to search for chemosensitizing compounds is the in vitro testing of the antifungal effect of putative sensitizers to determine their working concentration range and to make sure their toxicity is significantly lower than that of a fungicide used against the target pathogen. Recently, we found non-fungitoxic metabolites of plant origin, which enhanced the sensitivity of five cereal pathogens to one of DMI-fungicides [27]. Earlier, we also screened a range of microbial metabolites for sensitizing activity and briefly reported that a secondary metabolite of *Penicillium citrinum*, 6-demethylmevinolin (6-DMM) was the most promising as a putative sensitizer among them. 6-DMM was much less fungitoxic compared to tebuconazole, and it enhanced the sensitivity of a wheat and barley pathogen *B. sorokiniana* to one of tebuconazole-based fungicides [25]. We supposed that 6-DMM might be applied to improve the protective effect of triazoles against other soil or foliar pathogenic fungi attacking plants under field conditions, in the case that 6-DMM does not possess narrow antifungal and sensitizing activities towards a single pathogen. Therefore, the current study is the first step towards confirming this assumption and it aims to evaluate the effect of 6-DMM on the in vitro growth and germination of some other triazole-controlled pathogenic fungi in order to determine non- or marginally toxic 6-DMM concentrations for these species and to compare their sensitivity to this putative sensitizer. Here, we focused mainly on a 6-DMM activity towards *Fusarium* species (*F. avenaceum, F. culmorum, F. graminearum, F. oxysporum*), which most often form the common pathogenic complex of wheat or barley root rots together with *B. sorokiniana* [28,29,30]. Additionally, the effect on other important crop pathogens, such as *Alternaria alternata*, *A. solani*, causing early bight of tomato and potato, *Parastagonospora nodorum*, a causative agent of wheat glume/leaf blotch and *Rhizoctonia solani*, potato stem canker and black scurf agent, was tested. We also demonstrated 6-DMM sensitized *B. sorokiniana* to tebuconazole, and *R. solani* to azoxystrobin formulated as Folicur^®^, EC 250 and Quadris^®^, SC 250, respectively. 

## 2. Results

### 2.1. The Effect of 6-DMM on the Fungal Growth and Spore Germination

**Figure 1:**
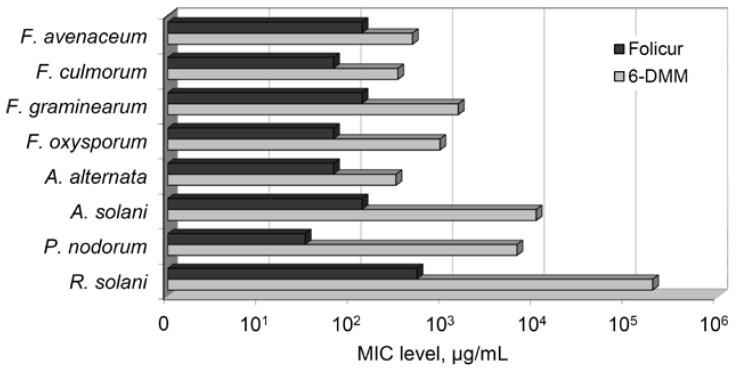
Minimum inhibitory concentrations (MIC) of 6-demethylmevinolin (6-DMM) and a tebuconazole-based fungicide (Folicur^®^ EC 250) preventing visible growth of fungal colonies.

The growth-inhibitory effect of 6-DMM towards several plant pathogens was evaluated after culturing the fungi on PDA containing 6-DMM at seven (*F. graminearum*), eight (*F. avenaceum*, *F. culmorum*), nine (*F. oxysporum*), or six (*A. alternata*, *A. solani*, *P. nodorum*, *R. solani*) concentrations that ranged, depending on the pathogen, from 5 to 800 µg/mL. Additionally, the conidia of some fungi were exposed to five (*Alternaria* spp.) or at least six (*Fusarium* spp.) 6-DMM concentrations varied in a wide range from 10 ng/mL to 15 µg/mL. As a result, nominally fungitoxic, sub-fungitoxic, and strongly or totally inhibiting concentration ranges causing 2–15%, 30–60% or 80–100% growth suppression, respectively, were selected for each pathogen. According to the obtained data, 6-DMM was found to affect both colony growth and conidial germination of all tested fungi. As in case of *B. sorokiniana* [25], this microbial metabolite was much less toxic for them than the tebuconazole-based fungicide (Figure 1). In our experiments, minimum inhibitory concentration (MIC) values of the fungicide exceeded those of 6-DMM for *F. avenaceum* and *F. culmorum* at least five-fold, and were either an order (*F. graminearum*, *F. oxysporum*, *A. alternata*) or several orders (*A. solani*, *P. nodorum*, *R. solani*) of magnitude higher. 

**Figure 2:**
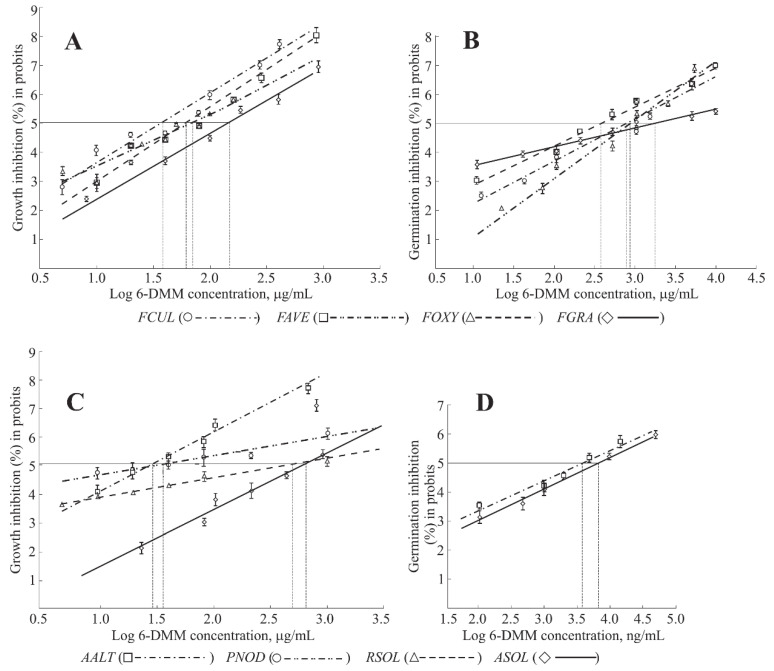
Dose-response curves showing inhibitory effect of 6-DMM on the growth (**A**,**C**) and germination (**B**,**D**) of various plant pathogenic fungi. FCUL, *Fusarium culmorum*; FAVE, *F. avenaceum*; FOXY, *F. oxysporum*; FGRA, *F. graminearum*; AALT, *Alternaria alternata*; PNOD, *Parastagonospora nodorum*; ASOL, *A. solani*; RSOL, *Rhizoctonia solani*. Vertical dotted lines cross the *X*-axis at the points corresponding to LogED_50_ for each of pathogens. Bars on the graphs (**A**–**D**) indicate SD and SE, respectively. The markers correspond to logarithms of the tested 6-DMM concentrations.

In general, 6-DMM concentrations causing 50% inhibition of *Fusarium* spp., *A. alternata*, and *P. nodorum* growth or their spore germination were significantly higher compared to ED_50_ for *A. solani* and *R. solani* (Table 1, Figure 2A,C).

Interestingly, ED_10_ values were almost equal for four *Fusarium* fungi, insignificantly differed in *A. alternata* or *P. nodorum* and did not exceed the minimum 6-DMM concentration previously found to enhance *B. sorokiniana* sensitivity to tebuconazole [25]. These data point to a common range of 6-DMM nominal toxic concentrations, which probably could be used in the studies on sensitization of all these cereal pathogens. 

When culturing on PDA supplemented with 6-DMM, *A. solani* manifested a much lower sensitivity to the putative sensitizer compared to *A. alternata*, for which the inhibitory effect of both nominally toxic and the growth suppressing 6-DMM concentrations was dozens of times higher as compared to *A. solani*. Besides, *A. solani* was completely insensitive to doses sensitizing *B. sorokiniana*. In contrast, no difference in the sensitivity to 6-DMM was found between these causative agents of Alternaria diseases in the spore germination tests (Table 1). Moreover, the dose-response patterns obtained for germinating conidia and colony growth of the pathogens were similar in both cases (Figure 2C,D).

With respect to the response to growth suppression, potato-damaging *R. solani* took almost the same position as the most insensitive *A. solani* (Table 1), at the same time showing the dose-response character similar to *P. nodorum* (Figure 2C).

### 2.2. The Sensitizing Effect of 6-DMM on B. sorokiniana and R. solani

For in vitro testing of the 6-DMM sensitizing activity, *B. sorokiniana* and *R. solani* were chosen, for which the maximum and minimum growth inhibition ED_95_ and MIC levels were determined (Table 1, Figure 1). 

**Figure 3:**
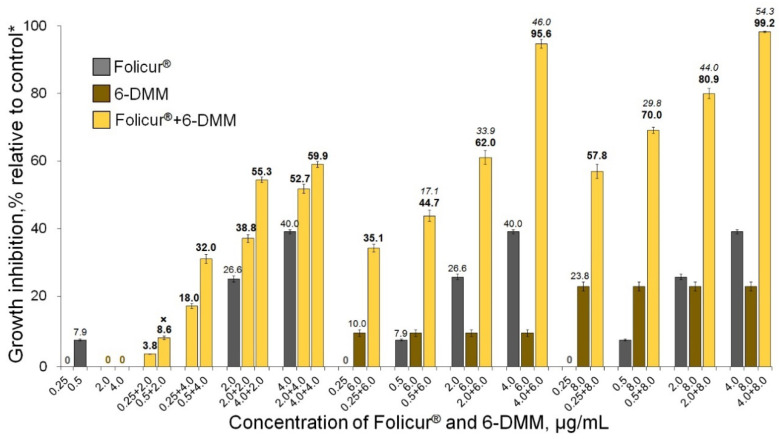
Enhancing the inhibitory effect of Folicur^®^ EC 250 on the in vitro growth of *Bipolaris sorokiniana* due the fungicide combination with 6-DMM. The numbers in bold or regular above the columns indicate *E_r_* values, while italic numbers show *E_e_* values related to the same fungicide/6-DMM concentration combination. *E_r_* shows the inhibition of the fungal growth (%) when the fungicide and 6-DMM were co-applied; *E_e_* is the inhibition calculated for an estimated additive effect of the fungicide and 6-DMM (%). In the case when *E_r_* > *E_e_* at *p* ≤ 0.05, a synergistic interaction between the fungicide and the sensitizer is confirmed (see Materials and Methods, Section 4.4 and [31]). The case of an additive effect is marked with “**×**”. Each histogram column represents the mean of three experiments (two diameter measuring of each colony, three colonies per each individual or combined application in each of three independent assays). Y-bars indicate standard error (SE) of the mean. The difference between treatments is significant at *p* ≤ 0.05 (*t*-test for independent variables). * Control colonies were cultured on potato dextrose agar free of 6-DMM and the fungicide.

**Figure 4:**
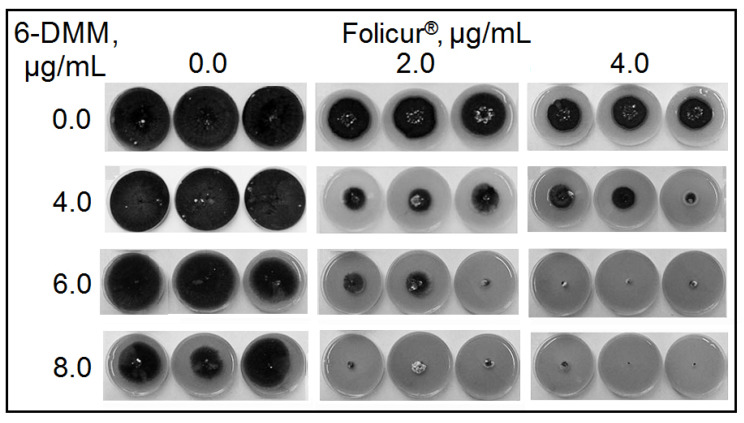
*Bipolaris sorokiniana* colonies grown for 9 days on potato dextrose agar supplemented with 6-DMM, Folicur^®^ EC 250, or their combinations. The shown Petri plate cultures represent a typical picture for one of three performed checkerboard assays.

**Figure 5:**
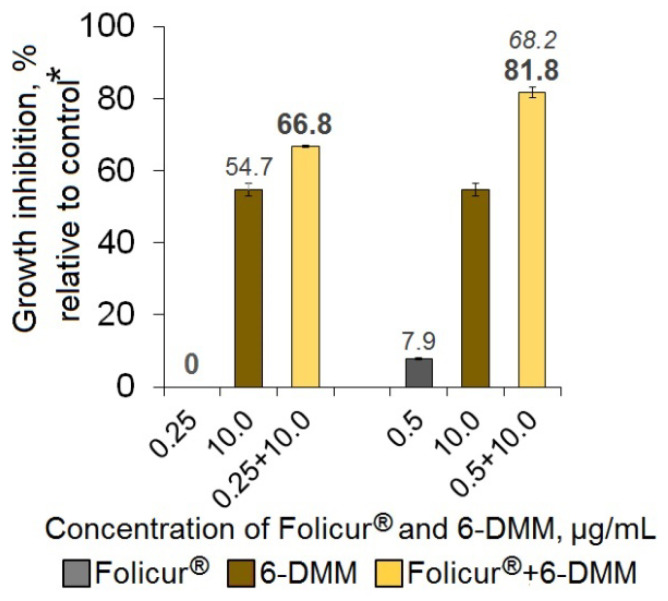
Synergistic augmentation of the in vitro suppression of the *Bipolaris sorokiniana* growth by ineffective Folicur^®^ doses after their combining with 6-DMM (10.0 µg/mL). Each bar represents the mean of three experiments. The italic number above the column indicates *E_e_*, other numbers indicate *E_r_* values; *E_r_* > *E_e_* at *p* < 0.05 (see Materials and Methods, Section 4.4). * Control colonies were cultured on potato dextrose agar free of 6-DMM and the fungicide.

**Figure 6:**
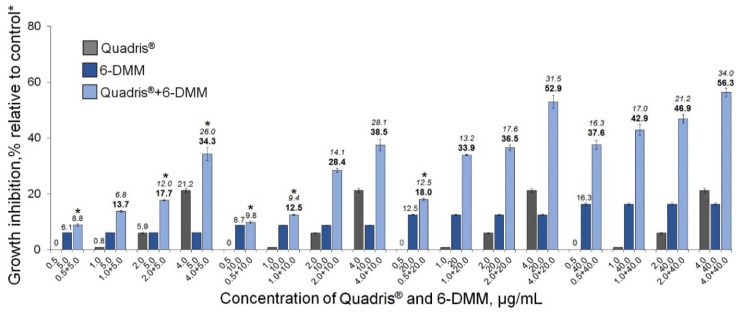
The inhibitory effect of Quadris^®^ SC 250 on the in vitro growth of *Rhizoctonia solani* augmented due the fungicide combination with 6-DMM. Each bar represents the mean of three experiments. Concentration combinations providing an additive effect are marked with asterisks; for other combinations, *E_r_* > *E_e_* at *p* ≤ 0.05 (see Materials and Methods 4.4). The difference between treatments is significant at *p* ≤ 0.05 (*t*-test for independent variables). * Control colonies were cultured on potato dextrose agar free of 6-DMM and the fungicide.

The pilot Petri plate bioassays involving co-applications of 6-DMM and Folicur^®^ or Quadris^®^ fungicides at different concentration combinations showed the inhibition of fungal growth to be significantly (*p* < 0.05) enhanced when both tested pathogens were cultured on fungicide-containing PDA amended with 6-DMM (Figure 3, Figure 4, Figure 5 and Figure 6). For *B. sorokiniana*, the synergistic interaction of 6-DMM and Folicur^®^ was observed in 15 of 32 concentration combinations tested by the checkerboard assay (Figure 3). In other cases, the effect was rather additive (*E_r_* ≥ *E_e_*, *p* > 0.05; data not shown). The most pronounced sensitizing effect was observed for 6-DMM concentrations equal to 4, 6, or 8 μg/mL applied together with Folicur^®^ at a ratio of 1.5:1, 2:1, or 4:1 (Figure 4). In these cases, the growth-inhibiting effect in relation to the pathogen demonstrated a 16-fold increase. For example, the MIC value of the Folicur^®^ used alone was 64 μg/mL, while, in the presence of the sensitizer, the complete inhibition of the fungal colony growth was observed at 4 μg/mL. The fractional inhibitory concentration indices (FICIs) varied from 0.27 to 0.36 (0.32 on average) confirming the synergetic interactions in these tebuconazole/6-DMM combinations and suggesting a significant increase in the *B. sorokiniana* sensitivity to the fungicide. A statistically significant excess of *E_r_* over *E_e_* evidencing the effect of synergism was revealed even for those combinations, where the 6-DMM dose reached 10 μg/mL (54.7% growth inhibition)**,** i.e., exceeded ED_50_ (Figure 5).

In the experiment with *R. solani*, the synergistic enhancement of the growth-inhibiting effect was also registered for the 6-DMM and Quadris^®^ (azoxystrobin) combinations (Figure 6). The *E_r_* values significantly exceeded *E_e_* values in 10 of 16 combinations tested, while another six variants of the combined use demonstrated either an additive effect, or a less than 10% increase in the inhibiting action of one of the components. 

## 3. Discussion

The antifungal effect of 6-DMM was evaluated using different plant pathogenic fungi, such as wheat pathogens *A. alternata*, *F. avenaceum, F. culmorum, F. graminearum*, and *P. nodorum*; tomato/potato pathogen *A. solani* causing early bight of these two crops, *F. oxysporum* infecting wheat and tomato, and *R. solani*, an important potato pathogen also able to infect a wide range of other crops. All these fungi are controlled with various triazoles, and tebuconazole is one of the most often applied among them [32]. The strains tested in our work demonstrated stable in vitro growth, active spore production, and the sensitivity to tebuconazole. Tebuconazole has been reported to show a good effect against a wide range of *Fusarium* species and other fungi during plant treatments, and its in vitro inhibitory effect has been well documented [33,34,35,36]. On the other hand, a widespread application of tebuconazole-containing formulations may promote selection and accumulation of tebuconazole-resistant strains in fungal populations [34,37,38] that decreases or even nullifies the sensitivity to tebuconazole resulting in in vitro variability of its fungicidal activity [39]. 

*Fusarium* species included in this study form a wheat root rot pathogenic complex able to cause significant yield losses in cereals across the world [29]. Since Fusarium root rot belongs to seed-transmitted diseases, seed treatment with fungicides still remains the main way to control it in spite of reports about the appearance of fungicide-resistant strains of target pathogens. In this context, the development of approaches providing the reduction in fungicide dosages without decreasing its antifungal effect is a very important practical task. The results of this study allowed us to determine the range of non- or marginally toxic 6-DMM concentrations for the studied *Fusarium* species. All four species were less sensitive to 6-DMM than *B. sorokiniana* at high growth-inhibiting concentrations and differed by the sensitivity to this microbial compound. However, high reliability of the approximation in the probit analysis confirmed with the R^2^ level determination (Table 1), similar dose-response pattern for 6-DMM in all four species (Figure 2A), and lower ED_10_ values (as compared to the dosage providing sensitizing effect for *B. sorokiniana*; Table 1) may evidence that 6-DIMM is a putative sensitizer to triazole fungicides for *Fusarium* pathogens and that it will be possible to select a working concentration of this compound providing an increased sensitivity of all components of the wheat root rot complex towards triazole fungicides.

*A. solani*, a foliar pathogen of tomato and potato, is considered to be poorly controlled by chemicals [40], while *R. solani* is a great problem in potato tuber storage. Early bight of tomato caused by *A. solani* is one of the most devastating diseases of this crop, while *A. alternata* is a soilborne fungus that often accompanies both Fusarium root rot agents, and *A. solani* on potato and tomato. *R. solani* on potato is often controlled by azoxistrobin and other strobilurins. These QoI-fungicides belong to the antifungal compounds with a high risk of resistance development in pathogens, and strobilurin-resistant *R. solani* strains have been reported for many pathogenic populations [8,11,41,42]. Our pilot findings on enhancing the sensitivity of these agents to Quadris^®^ open an avenue to further investigations on the chemosensitization of such resistant strains.

We found that strains pathogenic to *Solanum* plants were much less sensitive to the growth-suppressing action of 6-DMM as compared to strains of cereal pathogens (Table 1, Figure 2A,C). Additionally, a drastic distinction in the 6-DMM effect on the growth of *A. solani* and *A. alternata* was observed. In contrast, the germination of *A. solani* conidia was inhibited by 6-DMM as effectively as in *A. alternata* and other pathogens. 

Like many other known chemosensitizers, which generally have a mild antimicrobial activity and lack specificity to fungi [20], 6-DMM was revealed to possess such properties. Summing up all the presented results, one can conclude that they confirm a low fungitoxicity of 6-DMM, pointing to a wide spectrum of its antifungal activity, and suggesting that 6-DMM may have promise as a natural remedy for combination with chemicals to reduce their content in fungicidal formulations and their xenobiotic impact in agriculture, and probably, to enhance the protective effect of chemical fungicides by sensitization of various plant pathogens. 

*Fusarium*, *Alternaria*, and *Rhizoctonia* fungi produce a range of highly toxic secondary metabolites including trichothecene and polyketide mycotoxins [43,44]. These compounds cause mycotoxicoses in animals and are carcinogens and/or allergens for humans, while their producers are among nosocomial infections that are especially dangerous for immunodeficient patients. Earlier we showed that 6-DMM suppressed the biosynthesis of a polyketide aflatoxin B1 in a toxigenic *Aspergillus flavus* [45]. In this regard, there is a suggestion that the antifungal effect of 6-DMM may be accompanied by the inhibition of the biosynthesis of toxins in other toxigenic fungi. In this case, this putative sensitizer will have an additional beneficial effect.

The pilot studies on the assessment of the sensitizing effect of 6-DMM presented in this study first confirmed a principal possibility of using 6-DMM for the sensitization of *B. sorokiniana* and *R. solani* to the fungicides based on triazole (Folicur^®^) and strobilurin (Quadris^®^), respectively, which are widely used for the crop protection against these dangerous plant pathogenic fungi. A number of concentration combinations resulting in a synergistic enhancement of the in vitro fungicidal effect were revealed for both pathogens. Being less responsive to 6-DMM alone (Table 1), *R. solani* was also less responsive to sensitization by this agent compared to *B. sorokiniana*, and the higher doses of the sensitizer were needed to synergistically augment the Quadris^®^ effect against *R. solani*. Nevertheless, the results obtained for the fungicide/sensitizer co-applications at the most effective concentration combinations towards *B. sorokiniana* (4.0 + 8.0 μg/mL of Folicur^®^ + 6-DMM) and *R. solani* (4.0 + 40.0 μg/mL of Quadris^®^ + 6-DMM) suggest some prospects for 6-DMM combined with antifungal compounds of a different chemical nature and mode of action to improve their inhibitory effect. These findings also confirm the ability of 6-DMM to enhance the sensitivity of fungi belonging to different taxa, infecting different crops, and considerably differed in their pathogenesis processes.

The study on a sensitization of other plant pathogenic fungi with 6-DMM is in progress. The further investigations of this microbial product could contribute to overcoming the resistance of plant pathogens to agricultural fungicides and the reduction in the fungicide contamination of agricultural areas and products, as well as the total environmental pollution with toxic xenobiotics. 

## 4. Materials and Methods 

### 4.1. Fungal Strains and Their Culturing

Pathogen strains *A. alternata* MRD1-12, *Bipolaris sorokiniana* Ir-01-38, *F. avenaceum* Br-04-60, *F. culmorum* OR-02-37, *F. graminearum* FG-30, *F. oxysporum* KF-1713-4, and *R. solani* 100,063 isolated from various agricultural plants were provided by the State Collection of Plant Pathogenic Microorganisms of the All-Russian Research Institute of Phytopathology (ARRIP). The strains *A. solani* MO-VNIIF-9-2018 and *P. nodorum* B-9/47 were provided by the ARRIP work collections. 

Plant pathogens’ stock cultures maintained on potato dextrose agar (PDA) slants were resumed by culturing for 5–15 days (depending on the growth rate of a certain fungus) in Petri plates on the same medium to obtain spore-producing actively growing colonies. The aerial mycelia of these colonies at the log growth stage were used in the further experiments for inoculations of the fresh control or 6-DMM-containing PDA by placing a piece of the mycelium into the center of a 90-mm Petri plate. Conidia of *Fusarium* and *Alternaria* fungi were collected from a colony surface by flooding the mycelium with sterilized water and gently rubbing with a glass rod. Spore suspensions were filtered through sterile cotton wool to remove mycelial debris, and spore concentrations were counted using a hemocytometer. To simulate spore production in *Alternaria solani*, its cultures were flooded with sterilized ice water, irradiated with UV-A (315–360 nm) [46]. The Petri plates with treated colonies were allowed to dry for 3 days at room temperature under a diffused light [47] in sterilized laminar boxes followed by incubation at 24 °C for 7 days. 

### 4.2. Preparation of 6-DMM and Fungicide Samples for Microbiological Experiments

Since 6-DMM lactone produced by the microbiological synthesis and extracted from *P. citrinum* culture liquid [48] was insoluble in aquatic media, we transformed it into a water-soluble sodium salt prior to addition to the nutrient media. To do this, a preparation of 6-DMM was dissolved in hot ethanol, the ethanol solution was amended with an equimolar amount of NaOH, incubated for 30 min at gently mixing and diluted with distilled water so that the final 6-DMM content was not lower than 10% in order to prevent a pellet formation during storage that occurred in less concentrated solutions. A 6-DMM concentration in the resulting sample was determined by HPLC as described earlier [48]. Just prior to microbiological tests, portions of this sample were diluted with ethanol to prepare 1% 6-DMM stock solution, whose aliquots were added aseptically into sterilized warm melted PDA up to necessary concentrations before PDA inoculation with the pathogens. 

Commercial samples of Folicur^®^, EC 250 (a.i. tebuconazole) as well as Quadris^®^ SC 250 (a.i. azoxystrobin) used in sensitization tests with *B. sorokiniana* and *R. solani*, respectively, were dissolved in distilled water and sterilized by filtration through a 0.22-µm Millipore membrane. The minimal volumes of the stock filtrate were added to PDA as described above. 

### 4.3. In Vitro Assessment of a 6-DMM Effect on the Fungal Growth and Spore Germination

The influence of 6-DMM on the fungal growth was studied by a Petri plate bioassay involving the cultivation of fungi on PDA supplemented with different 6-DMM concentrations. The tested fungi were grown at 25–27 °C in the dark until the control colonies grown on 6-DMM-free PDA completely covered the agar surface. A growth inhibitory effect was determined by diminishing the average colony diameter measured on 6-DMM-containing PDA as compared to the control that was calculated after measuring the minimum and maximum diameters of colonies (six replications per treatment for each pathogen) in two perpendicular directions. 

To assess the effect of 6-DMM on the germination of *Fusarium* spp. and *Alternaria* spp., a microplate test [23,49] was applied. Briefly, fungal conidia were incubated for 5–6 h at room temperature in distilled water (control) or in aquatic solutions supplemented with the 6-DMM sodium salt. The number of germinated conidia (among 500 ones of each pathogen per treatment) was counted in the control and 6-DMM-containing spore suspensions using an inverted microscope followed by calculation of the percentage of conidia germination inhibition as compared to the control.

### 4.4. In Vitro Assessment of a 6-DMM Sensitizing Effect

The sensitization experiments were designed under the principle of a double-dilution test and a checkerboard assay [20,50,51]. Marginally fungicidal or sub-fungicidal concentrations of the fungicides and non-fungitoxic or marginally toxic doses of the putative sensitizer were selected in preliminary experiments including the pathogen cultivation in the presence of either 6-DMM or each fungicide. Then, the fungi were grown as described above on PDA supplemented with marginally fungicidal or sub-fungicidal concentrations of Folicur^®^ (*B. sorokiniana*) or Quadris^®^ (*R. solani*) causing 0–10% or 11–40% colony growth inhibition, respectively, and on PDA with 6-DMM in the concentration range of 2–10 µg/mL (*B. sorokiniana*) or 5–40 µg/mL (*R. solani*), which provided no or marginally toxic effect. In parallel, the fungi were grown on PDA containing both fungicide and 6-DMM taken at the same concentrations as alone. Control cultures were grown under the same conditions on PDA without any supplements. Except for the cases where both the sensitizer and the fungicides showed no detectable suppression of a fungal growth, the synergy in 6-DMM/fungicide combinations was determined by the Limpel formula [31]:

$$E_{e}=\frac{\left(X+Y\right)-XY}{100}<E_{r}\;(p\leq 0.05),$$

where *E_e_* is the expected additive inhibiting effect of the use of both components (%), *X* and *Y* represent the level of a spore germination inhibition by each of the components alone (%), and *E_r_* is the inhibition obtained by a joint use of 6-DMM and a fungicide. In addition, fractional inhibitory concentration indices (FICIs) [20,50] were calculated; according to generally accepted protocols, FICIs ≤ 0.5 were interpreted as a confirmation of synergistic interactions [50,52] between 6-DMM and a fungicide. 

### 4.5. Data Analysis and Statistical Treatment

Average 6-DMM inhibitory concentrations capable of 10, 50 or 95% suppression of the mycelium growth or spore germination (ED_10_, ED_50_, and ED_95_, respectively) were determined by the probit analysis [53] with the involvement of a linear regression based on the data obtained in two independent experiments for each pathogen. In a regression equation (*Y* = *ax*_n_ + *b*) used in probit-analyses, *Y* is the probit value for 10, 50 or 95% growth inhibition, *x* values represent logarithms of these EDs, while *a* and *b* represent regression coefficients. Minimum inhibitory concentrations (MIC), i.e., the lowest 6-DMM or fungicide concentrations preventing a visible colony growth, were detected by serial two-fold dilutions of the stock solutions according to a reference CLSI method for antifungal susceptibility testing of filamentous fungi [54]. Approximation confidence values (R^2^), regression coefficients, mean values, standard deviations, and standard errors as well as significant differences (at *p* ≤ 0.05) between treatments and the control, determined based on the *t*-test for independent variables, were calculated using a STATISTICA 6.0 package (StatSoft Inc., Tulsa, OK, USA).

## Figures and Tables

**Table 1:** Inhibitory concentrations of 6-dimethylmevinolin (6-DMM) for eight plant pathogenic fungi calculated by a probit analysis.

Pathogen	Inhibitory Concentrations of 6-DMM *						
	Colony Growth				Spore Germination		
	ED_10_, µg/mL	ED_50_, µg/mL	ED_95_, mg/mL	R^2^	ED_50_, µg/mL	ED_95_, mg/mL	R^2^
B. sorokiniana **	6.0 ^a^	9.5 ^a^	0.08 ^a^	0.977	1.30 ^a^	4.8 ^a^	0.939
*F. culmorum*	1.0 ^b^	38.0 ^b^	0.17 ^a^	0.923	0.35 ^b^	3.2 ^b^	0.917
*F. avenaceum*	1.3 ^b^	57.5 ^c^	0.26 ^b^	0.940	0.40 ^b^	4.1 ^a^	0.986
*F. oxysporum*	1.1 ^b^	63.1 ^c^	0.55 ^c^	0.932	0.80 ^c^	4.7 ^a^	0.956
*F. graminearum*	1.6 ^c^	141.3 ^d^	0.74 ^d^	0.973	1.25 ^d^	7.1 ^c^	0.913
*A. alternata*	4.8 ^a^	30.9 ^b^	0.17 ^a^	0.979	3.60 ^e^	5.2 ^d^	0.964
*A. solani*	85.8 ^d^	401.2 ^e^	13.16 ^d^	0.891	3.85 ^e^	5.4 ^d^	0.991
*P. nodorum*	7.1 ^a^	36.3 ^b^	7.59 ^e^	0.946	nd ***	nd	nd
*R. solani*	50.0 ^d^	398.0 ^e^	50.12 ^f^	0.883	nd	nd	nd

* Different uppercase letters within the column indicate statistically significant differences (*p* ≤ 0.05). ** For *B. sorokiniana*, ED_10_ is the minimum 6-DMM concentration determined experimentally, which provided effective sensitization of this pathogen to tebuconazole [25], while ED_50_ and ED_95_ were calculated in this work, using previously obtained data. ED means effective dose (see 4.5). *** Not determined.
